# Efficacy and safety of perioperative application of ketamine on postoperative depression: A meta-analysis of randomized controlled studies

**DOI:** 10.1038/s41380-023-01945-z

**Published:** 2023-01-20

**Authors:** Jie Guo, Di Qiu, Han-wen Gu, Xing-ming Wang, Kenji Hashimoto, Guang-fen Zhang, Jian-jun Yang

**Affiliations:** 1 Department of Anesthesiology, Shandong Cancer Hospital and Institute, Shandong First Medical University and Shandong Academy of Medical Sciences, Jinan, Shandong China; 2 Department of Anesthesiology, Pain and Perioperative Medicine, The first Affiliated Hospital of Zhengzhou University, Zhengzhou, Henan China; 3 Division of Clinical Neuroscience, Chiba University Center for Forensic Mental Health, Chiba, 260-8670 Japan; 4 Department of Anesthesiology, Shandong Provincial Hospital Affiliated to Shandong First Medical University, Jinan, China

**Keywords:** Depression, Predictive markers

## Abstract

Ketamine, a commonly used general anesthetic, can produce rapid and sustained antidepressant effect. However, the efficacy and safety of the perioperative application of ketamine on postoperative depression remains uncertain. We performed a meta-analysis to determine the effect of perioperative intravenous administration of ketamine on postoperative depression. Randomized controlled trials comparing ketamine with placebo in patients were included. Primary outcome was postoperative depression scores. Secondary outcomes included postoperative visual analog scale (VAS) scores for pain and adverse effects associated with ketamine. Fifteen studies with 1697 patients receiving ketamine and 1462 controls were enrolled. Compared with the controls, the ketamine group showed a reduction in postoperative depression scores, by a standardized mean difference (SMD) of −0.97, 95% confidence interval [CI, −1.27, −0.66], *P* < 0.001, I^2^ = 72% on postoperative day (POD) 1; SMD−0.65, 95% CI [−1.12, −0.17], *P* < 0.001, I^2^ = 94% on POD 3; SMD−0.30, 95% CI [−0.45, −0.14], *P* < 0.001, I^2^ = 0% on *P*OD 7; and SMD−0.25, 95% CI [−0.38, −0.11], *P* < 0.001, I^2^ = 59% over the long term. Ketamine reduced VAS pain scores on POD 1 (SMD−0.93, 95% CI [−1.58, −0.29], *P* = 0.005, I^2^ = 97%), but no significant difference was found between the two groups on PODs 3 and 7 or over the long term. However, ketamine administration distinctly increased the risk of adverse effects, including nausea and vomiting (risk ratio [RR] 1.40, 95% CI [1.12, 1.75], *P* = 0.003, I^2^ = 30%), headache (RR 2.47, 95% CI [1.41, 4.32], *P* = 0.002, I^2^ = 19%), hallucination (RR 15.35, 95% CI [6.^2^4, 37.34], *P* < 0.001, I^2^ = 89%), and dizziness (RR 3.48, 95% CI [2.68, 4.50], *P* < 0.001, I^2^ = 89%) compared with the controls. In conclusion, perioperative application of ketamine reduces postoperative depression and pain scores with increased risk of adverse effects.

## Introduction

Ketamine has been a commonly used general anesthetic in clinical practice for nearly 60 years [1]. It is mainly used for the induction and maintenance of anesthesia, sedation, and analgesia [2–5]. Outside of these properties, ketamine has also been found to be effective in managing depression, which has always been an important research topic in the past decade. In 2000, a study from Yale University first reported that a single intravenous (IV) injection of ketamine (0.5 mg/kg) exerted rapid-onset and sustained antidepressant effect in patients with depression [6], and several subsequent clinical studies further confirmed ketamine’s robust antidepressant effects in patients with treatment-resistant depression [7–12]. Additionally, accumulating evidence has indicated that ketamine rapidly decreases suicide ideation in patients with severe depression [13], which provides a foundation for perioperative administration of ketamine to improve postoperative depression.

Surgical treatment is a great challenge to patients and can induce psychological stress reactions, including anxiety and depression, during the perioperative period that affect postoperative recovery quality and may even increase postoperative complications [14, 15]. Approximately 10–30% of patients experience a depressed mood during the perioperative period [16], especially in patients undergoing cardiac surgery [17]. Patients with underlying depression preoperatively could have worsened the severity of depression after surgery [18, 19]. Additionally, postoperative depression has been described as a significant contributor to postoperative pain, decreased cognitive function, prolonged hospitalization, and morbidity [20–22]. Therefore, it is very valuable to investigate how to improve and prevent postoperative depression in patients undergoing surgery.

However, with related studies growing recently, the effect of ketamine on perioperative depression among surgical patients is still controversial. Several clinical trials have demonstrated that the perioperative application of ketamine was associated with improved postoperative depression scores, while some studies showed no significant antidepressant effects [23, 24]. Therefore, we performed a meta-analysis of the antidepression effects and related adverse effects of ketamine during the perioperative period to provide a clinical reference.

## Methods

The meta-analysis was conducted in accordance with Preferred Reporting Items for Systematic Reviews and Meta-Analyses guidelines [25]. This study was conducted in accordance with an established protocol and prospectively registered in the international prospective register of systematic reviews (PROSPERO) [26], and the registration information is available at https://www.crd.york.ac.uk/PROSPERO/ (registration number: CRD42020185268).

### Literature search and screening

**Fig. 1:**
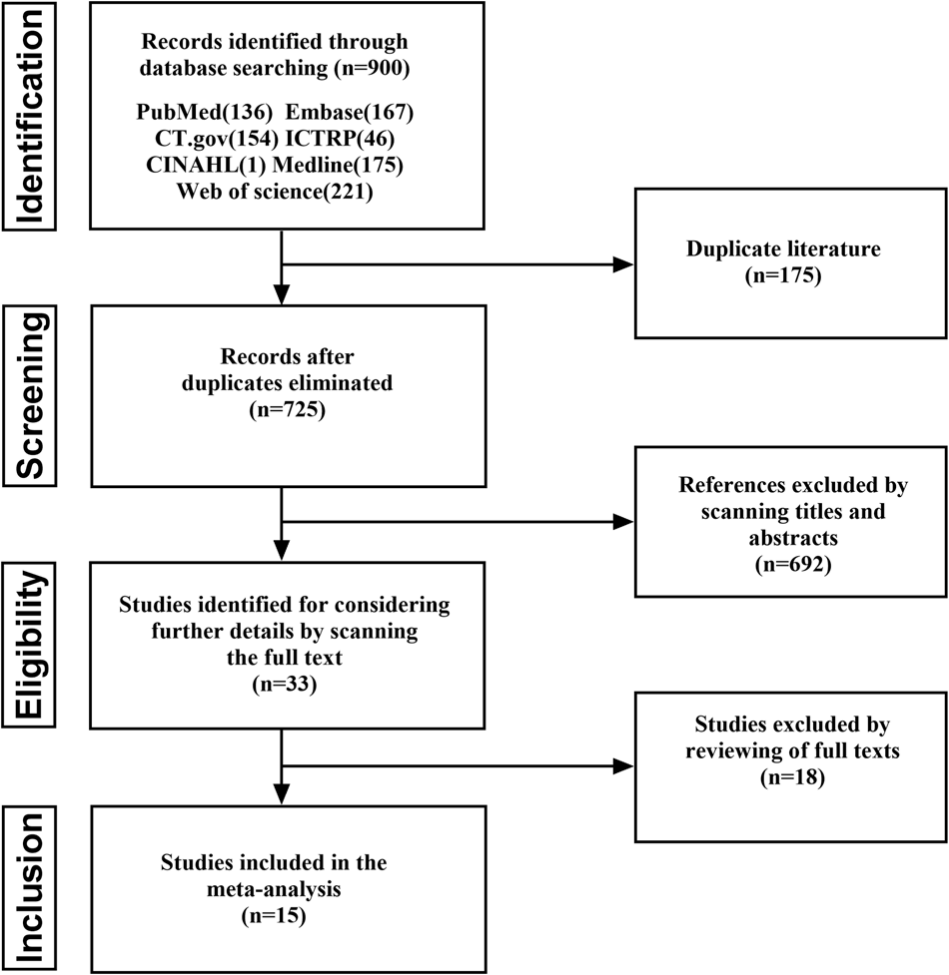
Flow chart for study selection. Total number of studies identified, screened, deemed eligible, and ultimately included is summarised.

We performed a systematic literature search using the key words “(ketamine OR N-methyl-D-aspartic acid OR NMDA OR glutamate) AND (depression OR depressive OR depressed OR mood) AND (perioperative OR anesthesia OR surgery OR perioperative)” with the limitations “English written”, “clinical trial”, and “randomized controlled trial” in the Cochrane Central Register of Controlled Trials (CENTRAL, including PubMed, Embase, CINAHL, Clinical trials, and the WHO’s International Clinical Trials Registry Platform), Medline, and Web of Science. The search included dates through May 22, 2022, and was conducted by two independent investigators (the initial two authors: J.G. and D.Q.). In the initial screening stage, both investigators screened the titles and abstracts of all articles. Inconsistent selections and disagreements were discussed to reach a consensus. In the subsequent stage of screening for eligibility, the inclusion criteria were as follows: (1) randomized studies that explored the perioperative application of ketamine (experimental group) in comparison with a control group (saline or other drugs) for postoperative depressed mood; and (2) articles on human clinical trials. Articles were excluded if they were (1) reviews, case reports, or nonrandomized studies or (2) included no experimental or control group or the relevant data on the interesting outcomes could not be extracted. The selection protocol is depicted in Fig. 1.

### Data extraction

We extracted data from the included studies using Microsoft Excel and then transcribed the data to Review Manager (Version 5.4) for statistical analysis. The study characteristics analyzed included author name, study design, number of patients, surgical type, anesthesia type, time and dose of ketamine intervention, postoperative depression score, pain intensity score, postoperative adverse effects, and follow-up period. We extracted all rating scale scores regarding changes in depressed mood from the included studies as the primary outcome, including the Patient Health Questionnaire (PHQ), Montgomery-Åsberg Depression Rating Scale (MADRS), Beck Depression Inventory (BDI), Hospital Anxiety and Depression Scale (HADS), Profile of Mood States (POMS), and Edinburgh Postnatal Depression Scale (EPDS) scores. The secondary outcomes were the postoperative visual analog scale pain scores and adverse effects (nausea and vomiting, headache, hallucination, and dizziness). When data were not available in the articles, we attempted to contact the authors to request the original data or use Get Data software to obtain the original data from the figure. Furthermore, if multiple rating scales were used in one study, we gave preference to the MADRS. Four treatment time points were chosen (postoperative days 1, 3, and 7 and over the long term) to assess the effects of ketamine. If multiple time points were tested in one study at 7 days postoperatively, a time point close to 1 month was preferentially selected. Additionally, the study quality and risk of bias were independently assessed by two trained reviewers according to the Cochrane Collaboration’s risk of bias tools [27] (Review Manager 5.4). The risk of bias in each trial was assessed into “low”, “high”, and “some concerns” based on the following domains: random sequence generation, allocation concealment, blinding of participants and personnel, blinding of outcome assessment, incomplete outcome data, selective outcome reporting, and other biases. Any discrepancies were resolved via discussion to reach a consensus.

### Subgroup analysis

To examine whether the observed effects of ketamine on postoperative depression varied in terms of the moderator variables, the following subgroup analyses were conducted based on the presence of depression preoperatively (with vs. without), type of anesthesia (spinal anesthesia vs. general anesthesia), method of ketamine administration (single-dose vs. continuous infusion administration), dose of ketamine (low vs. high dose) and midazolam premedication (with vs. without). We were unable to perform a subgroup analysis on the effect of (*S*)-ketamine (or esketamine) and ketamine because there were only one or two studies in each subgroup. To further investigate the safety of intraoperative ketamine for antidepressant use, we performed a subgroup analysis of ketamine-related adverse effects with regard to the method of ketamine administration (single vs. continuous infusion), dose of ketamine (low dose vs. high dose), type of anesthesia (spinal anesthesia vs. general anesthesia) and midazolam premedication (with vs. without).

### Statistical analysis

In the present study, the meta-analytic procedures consisted of the following three parts: (a) differences in a postoperative depressive mood, (b) differences in VAS scores, and (c) differences in treatment-related adverse effects. For the continuous variables, the standardized mean difference (SMD) with 95% confidence intervals (95% CIs) was calculated because different scales were employed between studies to measure the same outcome. Dichotomous variables were pooled as the risk ratio with 95% CI. If the trial did not report a particular continuous outcome in the form of a mean and standard deviation, we converted values according to a mature conversion method [28]. We assessed heterogeneity using the *I*^2^ value, which estimates the amount of total variation that is attributable to heterogeneity. If the values were <50%, a fixed effects model was chosen. If heterogeneity was >50%, we selected a random effects model and explored sources of heterogeneity using further a subgroup analysis. A two-tailed *p* value <0.05 was considered to indicate statistical significance.

## Results

### Study selection and characteristics

The initial internet search yielded 900 potentially relevant studies. After excluding duplicates, 725 studies remained. Among these studies, 692 studies were excluded after reviewing the titles and abstracts. Among the remaining 33 studies, 18 studies were excluded after the two reviewers independently examined the full texts for the following reasons: application of epidural ketamine, no relevant data regarding the postoperative depression outcome, and ongoing studies. Finally, 15 studies that compared ketamine with placebo fulfilled all inclusion criteria and were used to perform the meta-analysis **(**Fig. 1**)**. Altogether, 15 studies included 3159 adult patients undergoing surgical treatment [23, 24, 29–41]. Of the 15 eligible trials, there were nine studies from China, one from the USA, one international multicenter study (four countries and ten centers), and one each from Korea, Egypt, Iran, and Japan. Fourteen studies used saline as a control, and one study used dexamethasone as an active control. Four studies included patients with preoperative depression, eight studies excluded patients with preoperative depression, and three studies included patients with no restrictions. Anesthesia types included spinal anesthesia in five studies and general anesthesia in 10 studies. Additionally, four studies received a single dose of ketamine, and nine studies received continued infusion of ketamine. One study used ketamine in patient-controlled intravenous analgesia and one study used two combined methods. Eight studies received high-dose ketamine (≥0.5 mg/kg), and seven studies received low-dose ketamine (<0.5 mg/kg). The main characteristics of the included randomized controlled trials (RCTs) are described in Table 1.

**Table 1:** Characteristics of included studies.

First author	Year	Country	Study Design and sample size (T/C)	Age (T/C)	Sex	Surgery	Anesthesia	Ways of ketamine injection	Time point of intervention	Intervention (T vs. C)	Depression Measurement	Pain Measurement	Follow-up Period
Kudoh A	2002	Japan	RCT	46.9 ± 8.8/	M+ F	Orthopedic surgery	General anesthesia	Single dose	Induction	1.0 mg/kg IV KET vs. normal saline	HAMD	VAS	Post-op days 1 and 3
			70(35/35)	48.2 ± 7.4									
Jiang M	2016	China	RCT	43.4 ± 0.95/	M+ F	Orthopedic surgery	General anesthesia	Single dose+ Continuous infusion	Induction+ Intraoperative	0.5 mg/kg IV KET vs. normal saline and 0.25 mg/kg/h IV KET vs. normal saline for 30 minutes	PHQ-9	VAS	Post-op day 1 and 5
			120(60/60)	41.40 ± 0.16									
Xu Y	2017	China	RCT 330(165/165)	31 ± 4/32 ± 4	F	Cesarean section	Spinal anesthesia	Single dose	After clamping the neonatal umbilical cord	0.25 mg/kg IV KET vs. normal saline	EPDS	NRS	Post-op day 3 and week 6
Xu R	2017	China	RCT 50(25/25)	43.27 ± 6.6/	F	Breast cancer radical mastectomy	General anesthesia	Continuous infusion	1 h after induction of anesthesia	0.5 mg/kg IV KET for 10 mins vs. normal saline	HAMD	VAS	Post-op day 1,3 and 7
				42.36 ± 7.28									
Mashour GA	2018	International	RCT 670(226/223/221)	70 ± 7.2/	M+ F	Major surgery	General anesthesia	Single dose	Intraoperatively (After induction)	KET-Low (0.5 mg/kg) IV vs. KET-High (1.0 mg/kg) IV vs. normal saline	PHQ-8	/	Post-op day 3 and 30
				70 ± 7.2/									
				70 ± 6.9									
Lee C	2019	South Korea	RCT 297(99/99/99)	53.9 ± 9.7/	F	Laparoscopic gynecologic surgery	General anesthesia	Single dose	Intraoperatively	0.5 mg/kg IV KET vs. 0.1 mg/kg IV DEX vs. 0.5 mg/kg IV KET + 0.1 mg/kg IV DEX	PHQ-9	VAS	Post-op day 1 and 3
				54.1 ± 8.0/					(5 min after induction)				
				53.8 ± 7.3									
Ma JH	2019	China	RCT 686(343/343)	å 18	F	Cesarean Section	Spinal anesthesia.	Single dose	10 min after child birth	0.4 mg/kg IV KET vs. normal saline	EPDS	NRS	Postpartum day 4 and 42
Wang J	2019	United States	RCT	39.9 ± 30.7/	M+ F	Laparoscopic gastric	General anesthesia	Continuous infusion	PACU	0.4 mg/kg (by ideal body weight) IV KET over 20 min vs. normal saline	MADRS	VAS	Post-op day 1, 2 and 7
			90(44/46)	41.3 ± 34.4		bypass and gastrectomy							
Liu PR	2019	China	RCT 303(101/102/100)	46.6 ± 8.2/	F	Breast cancer	General anesthesia	Single dose	Intraoperatively (After induction)	0.125 mg/kg IV S-KET vs.0.125 mg/kg IV R-KET vs. normal saline	HAMD-17	VAS	Post-op day 3, 7 and week1, 3
				47.7 ± 9.7/									
				48.0 ± 10.2									
Yao JX	2020	China	RCT	30 ± 4/30 ± 3	F	Cesarean Section	Spinal anesthesia	Single dose	5 mins after clamping the neonatal umbilical cord	0.25 mg/kg IV KET vs. normal saline	EPDS	NRS	Post-op week1, 2 and month 1
			308 (153/ 155)										
Mostafa RH	2021	Egypt	RCT	28.7 ± 5.6/	F	Dilation and Curettage	Spinal anesthesia	Continuous infusion	Intraoperatively	0.4 mg/kg IV KET over 20 mins vs. normal saline	POMS	/	Post-op hour 2
			60 (30/30)	29.1 ± 7.5									
Zhou Y	2021	China	RCT	49.5 ± 16.1/	M+ F	Intracranial Tumor Resection	General anesthesia	Continuous infusion	Intraoperatively	0.5 mg/kg IV KET for 40 mins vs. normal saline	MADRS	NRS	Post-op day1, 2, 3 and at discharge
			84 (41/43)	47.4 ± 9.9									
Alipoor M	2021	Iran	RCT	27.4 ± 4.09/	F	Cesarean Section	General anesthesia	Single dose	Induction	0.5 mg/kg IV KET vs. normal saline	EPDS	/	Post-op week 2 and 4
			134(67/67)	28.24 ± 4.81									
Han YQ	2022	China	RCT 275(122/153)	31.64 ± 3.93/	F	Cesarean Section	Spinal anesthesia	PCIA	PCIA	Sufentanil 2 μg/kg + tropisetron 10 mg + 0.5 mg/kg S-ketamine vs. Sufentanil 2 μg/kg + tropisetron 10 mg	EPDS	VAS	Post op day 3, 14 and 28
				31.85 ± 4.16									
Ren Q	2022	China	RCT 104(26/26/26/26)	61.6 ± 4.5/62.9 ± 7.3/	M+ F	Colorectal surgery	General anesthesia	Single dose	5 min before operation	0.1 mg/kg IV KET vs.0.2 mg/kg IV KET vs.0.3 mg/kg IV KET vs. normal saline	HADS	VAS	Post op day 1,2 and 3
				60.5 ± 12/63.9 ± 4.7									

*EPDS* Edinburgh Postnatal Depression Scale, *HADS* Hospital Anxiety and Depression Scale, *HAMD-17* Hamilton Rating Scale for Depression–17 item, *MADRS* Montgomery–Åsberg Depression Rating Scale, *PHQ-9* Patient Health Questionnaire-9, *POMS* Profile of Mood States, *VAS* Visual Analog Scale.

### Quality assessment

**Fig. 2:**
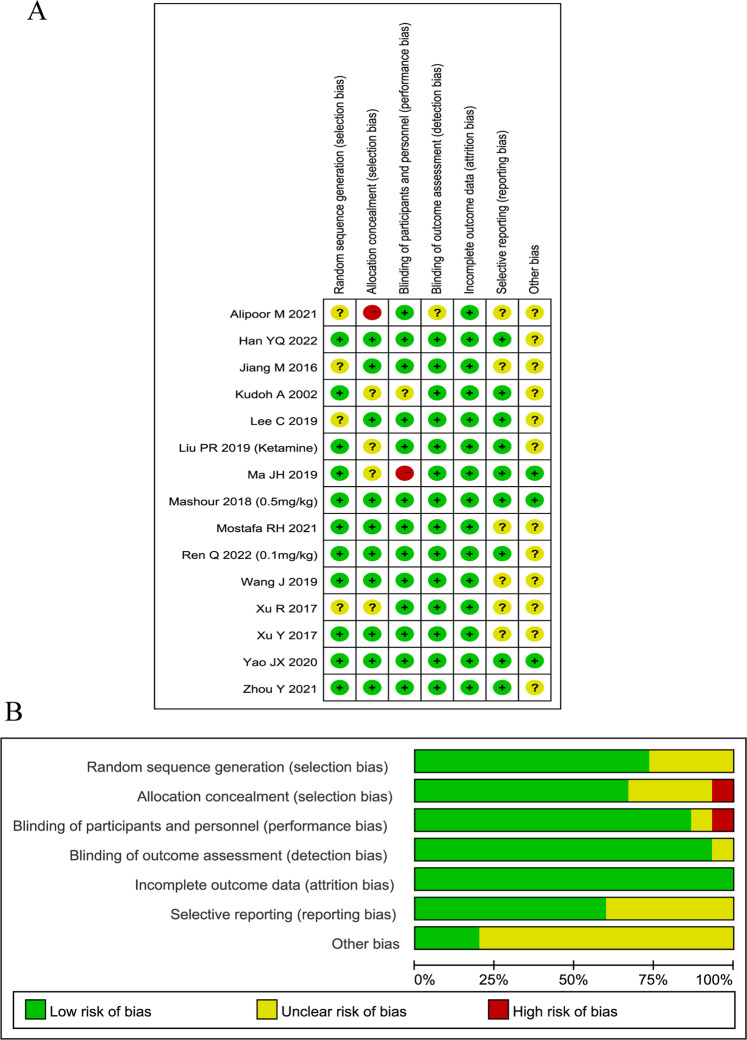
Risk of bias assessment. **A** Risk of bias for individual studies. **B** Risk of bias summary.

Of the included trials, four studies did not provide sufficient information regarding random sequence generation, and four studies did not provide adequate details on allocation concealment. One study did not blind the anesthesiologists, and one study did not provide information on the blinding of participants and personnel. We assessed the study quality and risk of bias in accordance with specific conditions. The risk of bias assessment for individual studies and its summary are presented in Fig. 2A, and B, respectively.

### Postoperative depression scores

**Fig. 3:**
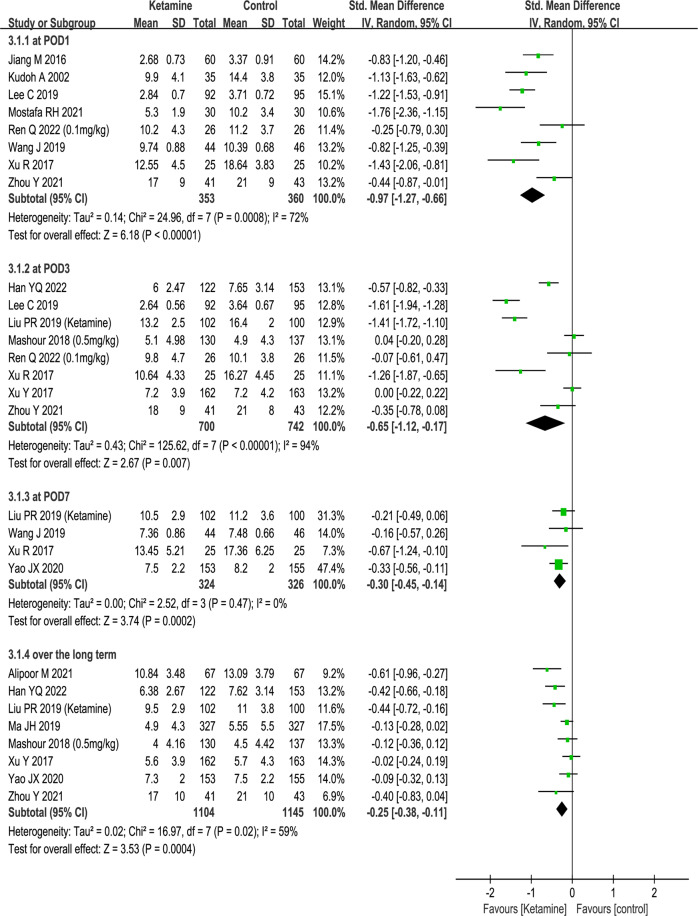
The effects of perioperative application of ketamine on postoperative depression. Forest plots of the postoperative depression rating scale in randomized controlled trials. Random-effects meta-analysis. POD postoperative day. CI confidence interval. df degrees of freedom.

The first part of our main meta-analysis investigated the different effects of ketamine and saline/dexamethasone on depression scores from surgical patients on PODs 1, 3, and 7 over the long term. Altogether, 1697 patients receiving ketamine/esketamine and 1462 patients receiving saline/dexamethasone as controls were extracted from the 15 recruited studies. The antidepressant effect was better in the ketamine group than in the control group. Compared with the placebo group, the ketamine group showed positive effects on POD 1 (SMD − 0.97, 95% CI [−1.27, −0.66], P < 0.001, I^2^ = 72%), POD 3 (SMD − 0.65, 95% CI [−1.12, −0.17], *P* < 0.001, I^2^ = 94%), POD 7 (SMD − 0.30, 95% CI [−0.45, −0.14], *P* < 0.001, I^2^ = 0%), and over the long term (SMD−0.25, 95% CI [−0.38, −0.11], *P* < 0.001, I^2^ = 59%). Forest plots of this analysis are presented in Fig. 3.

### Postoperative pain scores

**Fig. 4:**
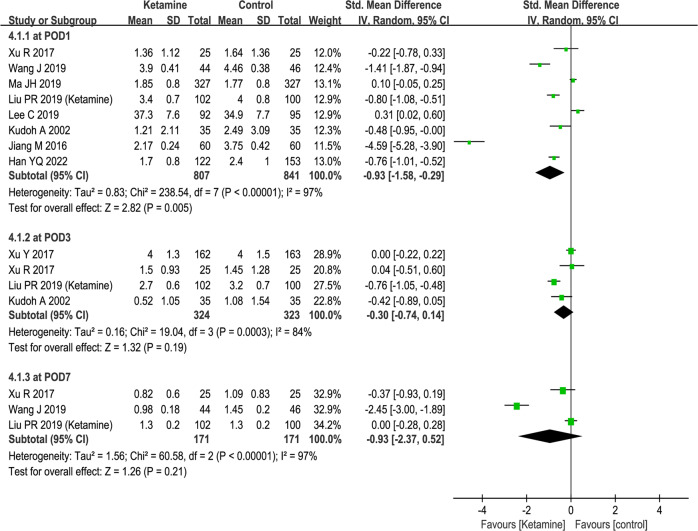
The effects of perioperative application of ketamine on postoperative pain. Forest plots of postoperative pain intensity in randomized controlled trials. **A** Postoperative pain intensity. Random-effects meta-analysis. POD postoperative day, CI confidence interval, df degrees of freedom.

In the second part of the meta-analysis, we compared the postoperative pain intensity assessed using VAS pain scores between patients receiving ketamine and those receiving control drugs. Our analysis indicated that ketamine was more effective than the placebo in reducing postoperative pain intensity on POD 1 (SMD − 0.93, 95% CI [−1.58, −0.29], *P* = 0.005, I^2^ = 97%), whereas no significant difference was observed in the ketamine group on POD 3 (SMD − 0.30, 95% CI [−0.74, −0.14], P = 0.19, I^2^ = 84%) and POD 7 (SMD − 0.93, 95% CI [−2.37, −0.52], *P* = 0.21, I^2^ = 97%). The forest plots of this analysis are presented in Fig. 4.

### Adverse effects

**Fig. 5:**
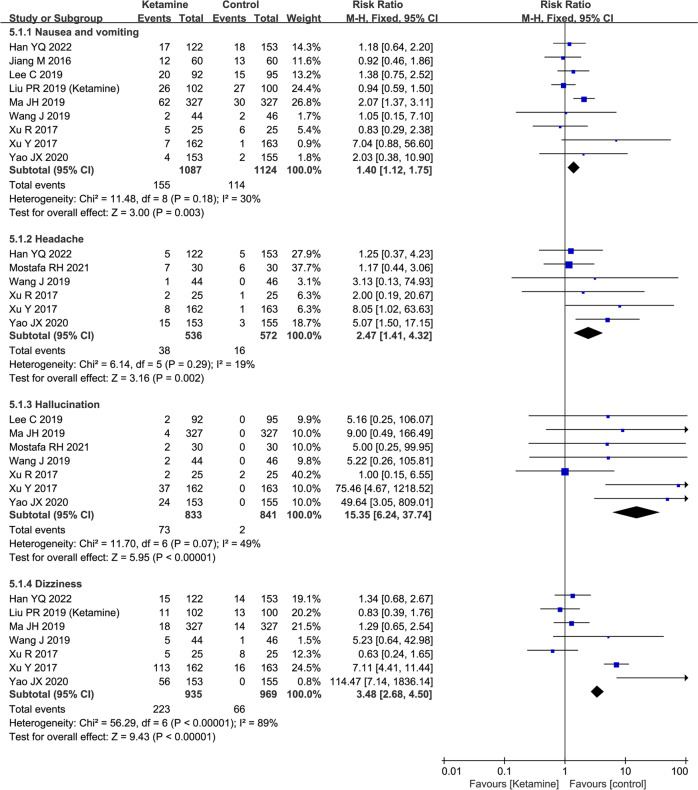
Forest plots of adverse effects in randomized controlled trials. Random-effects meta-analysis. POD postoperative day, CI confidence interval, df degrees of freedom. **A** nausea and vomiting. **B** headache. **C** hallucination. **D** dizziness. Random-effects meta-analysis. CI confidence interval. df degrees of freedom.

Next, we investigated the effects of ketamine on adverse reactions, including nausea and vomiting, headache, hallucinations, and dizziness. The ketamine group had a higher risk of nausea and vomiting (RR 1.40, 95% CI [1.12, 1.75], *P* = 0.003, I^2^ = 30%), headache (RR 2.47, 95% CI [1.41, 4.32], *P* = 0.002, I^2^ = 19%), hallucination (RR 15.35, 95% CI [6.24, 37.34], *P* < 0.001, I^2^ = 89%), and dizziness (RR 3.48, 95% CI [2.68, 4.50], *P* < 0.001, I^2^ = 89%) than the control group **(**Fig. 5**)**.

### Subgroup analysis

#### Postoperative depression scores

Subgroup analysis for postoperative depression scores between patients with and without preoperative depression showed that ketamine had beneficial effects on POD 1 on both patients with (SMD − 0.97, 95% CI [−1.56, −0.38], *P* = 0.001, I^2^ = 75%) and without preoperative depression (SMD − 1.01, 95% CI [−1.51, −0.50], *P* < 0.001, I^2^ = 81%) compared with placebo (Supplementary Fig. 1A). As most studies on postpartum depression focused on the long-term outcomes after operation, we performed a subgroup analysis of anesthesia type over the long term after operation. According to a subgroup analysis of the anesthesia type, ketamine reduced postoperative depression scores over the long term in patients with spinal anesthesia (SMD − 0.23, 95% CI [−0.41, 0.05], *P* = 0.01, I^2^ = 69%) and patients with general anesthesia (SMD − 0.29, 95% CI [−0.52, −0.07], *P* = 0.01, I^2^ = 41%) (Supplementary Fig. 1B**)**. Additionally, both single-dose administration and continued infusion of ketamine obviously relieved the postoperative depression scores on POD 1 (SMD − 0.89, 95% CI [−1.27, −0.50], *P* < 0.001, I^2^ = 70%; SMD − 1.08, 95% CI [−1.65, −0.51], *P* < 0.001, I^2^ = 80%) (Supplementary Fig. 2A**)**. Regarding the intervention dose, ketamine demonstrated remarkable beneficial effects on postoperative depression scores in the low-dose (<0.5 mg/kg) (SMD − 0.93, 95% CI [−1.71, −0.15], *P* = 0.02, I^2^ = 85%; 3 studies) and high-dose (≥0.5 mg/kg) groups (SMD − 1.04, 95% CI [−1.46, −0.62], *P*  < 0.001, I^2^ = 71%; 4 studies), as compared with placebo (Supplementary Fig. 2B). Subgroup analyses showed that midazolam did not affect the effects of ketamine on postoperative depression scores (Supplementary Fig. 3).

#### Adverse effects

Ketamine increased the risk of nausea and vomiting (Supplementary Fig. 4A), headache (Supplementary Fig. 4B), and hallucination (Supplementary Fig. 5A) compared with placebo in the single-dose groups. Contrarily, there were no significant differences when ketamine was continuously infused (Supplementary Figs. 4, 5). Additionally, ketamine increased the incidence of headache, hallucination, dizziness in patients without midazolam premedication, whereas no significant difference was found in patients with midazolam premedication (Supplementary Figs. 6B, 7A, B). However, there was no significant difference between ketamine group and control group in the incidence of nausea and vomiting whether midazolam premedication is used or not (Supplementary Fig. 6A). Subgroup analyses of adverse effects with regard to anesthesia type showed that ketamine increased the risk of nausea and vomiting (Supplementary Fig. 8A), hallucination (Supplementary Fig. 9A) and dizziness (Supplementary Fig. 9B) in spinal anesthesia group than general anesthesia group. There was no significant difference in the incidence of headache between ketamine group and control group (Supplementary Fig. 8B). These results suggest that continuous infusion administration of ketamine with midazolam or other general anesthetics could reduce the adverse effects of ketamine.

## Discussion

This meta-analysis of 3159 participants, including 1697 participants in the ketamine group and 1462 participants in the placebo group, showed that ketamine has a prophylactic effect on postoperative depression. The results can offer valuable evidence for making appropriate pharmacotherapy decisions in clinical practice to improve perioperative depression.

In this meta-analysis, ketamine improved the postoperative depression scores on PODs 1, 3, and 7 over the long term (up to the 42nd day postoperatively). Previous studies have shown that ketamine can produce rapid onset and sustained (>2 weeks) antidepressant effects in treatment-resistant patients with depression after a single-dose administration [42, 43], corroborating the findings of this review. However, the inconsistent results in the meta-analysis should not be ignored. An international, multicenter, double-blind, randomized study showed that intraoperative injection of ketamine did not prevent depression or decrease depressive symptoms after a major surgery in patients aged >60 years [23]. Additionally, Wang et al. [24] found that ketamine did not make a significant difference in postoperative depression scores, which could be attributed to the fact that patients in the study were healthier with quite low baseline MADRS or BDI scores compared with the patients in other studies. Additionally, the MADRS and BDI are more suitable for patients with serious depression rather than with mild depression. A further study is required to clarify whether ketamine might prevent postoperative depression.

The mechanisms underlying ketamine’s antidepressant effects have not been fully elucidated. Accumulating preclinical data suggest that neurotrophic and growth factors such as brain-derived neurotrophic factor (BDNF) and transforming growth factor β plays a key role in the antidepressant effects of ketamine and its enantiomers [42–46]. In clinical studies, Jiang et al. showed that the improvement in patients with postoperative depression after ketamine administration was associated with elevated serum levels of BDNF [29]. The antidepressant effect of ketamine may be linked to its immunomodulatory and anti-inflammatory effects, as the inflammatory cytokine levels are always increased in surgical patients [33, 47, 48]. Yang et al. reported that serum interleukin-6 could be a predictive biomarker for the antidepressant actions of ketamine in treatment-resistant patients with depression [49]. Furthermore, ketamine is increasingly used as an analgesic for perioperative pain. The link between depression and pain is bidirectional, and both act as risk factors for each other [50, 51]. The analgesic effect of ketamine may be associated with its antidepressant effect, as supported by our meta-analysis that showed ketamine reduced pain intensity on POD 1. Nonetheless, a further study on the underlying effect of ketamine on the link between depression and pain is needed. In addition to NMDA receptor, ketamine is known to interact with opioid receptors [52]. Although a clinical study using small sample size suggested the role of opioid receptor in the antidepressant effects of ketamine [53], the role of opioid receptor system is debatable [54, 55]. Given the analgesic effects of ketamine, it seems that opioid receptors may play a role in the mechanisms of action on postoperative pain. Nonetheless, further clinical study using opioid receptor antagonist is needed to ascertain the role of opioid receptor system in the mechanism of ketamine on postoperative depression. To further identify the population suitable for ketamine use, we conducted a subgroup analysis of the antidepressant effect of ketamine according to whether preoperative depression was present and the different anesthesia types. We found that there were no significant differences in ketamine’s antidepressant effect between patients with and without preoperative depression. Furthermore, ketamine had antidepressant effects on both patients who undergone surgery under spinal anesthesia and general anesthesia. Contrarily, a recent meta-analysis by Wang et al. [56] found that ketamine reduced the postoperative depression scores on POD 3 in patients with preoperative depression, whereas no significant difference was found in nonrestrictive studies. The mixed results may be explained by the latter meta-analysis including only two studies [23, 41].

It is not known whether the antidepressant effect of ketamine is affected by concomitant use of general anesthetics. It is difficult to analyze the effect of propofol, volatile anesthetics, and opioids alone on the antidepressant effect of ketamine because these anesthetics were used as most general anesthesia in this meta-analysis. As an alternative, we performed subgroup analyses with regard to spinal and general anesthesia to examine the total influence of general anesthetics. The results showed that there was no significant difference in antidepressant effect of ketamine between spinal anesthesia and general anesthesia. Furthermore, ketamine-related psychiatric adverse effects were observed when it was administered alone [57], but the concurrent administration of benzodiazepine can reduce these psychiatric adverse effects [58]. In this meta-analysis, the subgroup analysis showed that midazolam premedication could reduce the incidence of ketamine-related headache, hallucination, and dizziness. Collectively, the antidepressant effect of ketamine cannot be affected by general anesthetics such as propofol, volatile anesthetics and midazolam, but ketamine-related adverse effects can be ameliorated by midazolam.

The present meta-analysis revealed a higher risk of nausea and vomiting, headache, visual hallucinations, and dizziness in those receiving ketamine than in the controls. Almost all the previous studies have shown a rapid-acting antidepressant effect of ketamine (0.5 mg/kg over 40 min, IV); however, the optimal dose of ketamine for the treatment of depression remains unknown. A recent double-blind, placebo-controlled study using different doses of ketamine (0.1, 0.2, 0.5, 1.0 mg/kg, over 40 min, IV) demonstrated the antidepressant efficacy of high doses (0.5 and 1.0 mg/kg) of ketamine [59]. However, the high doses of ketamine caused more dissociative symptoms and elevated blood pressure than the low doses [59]. The ketamine dose administered in the included patients in the meta-analysis ranged from 0.1 to 1.0 mg/kg, the most commonly used dose was 0.5 mg/kg. It is generally believed that the adverse effects increase with increasing dose, but there was no significant difference in the adverse reactions between the high (≥0.5 mg/kg) and low-dose (<0.5 mg/kg) groups in the current meta-analysis. The possible reason is the multiple effects of anesthesia and surgery on patients, which still needs further study

Additionally, a subgroup analysis of safety found that continuous infusion of ketamine could effectively reduce the adverse effects related to ketamine compared with single-dose ketamine administration while maintaining its antidepressant effect. Thus, it is necessary to pay more attention to the optimal dose and approach of ketamine for postoperative depression. Notably, the (*S*)-enantiomer of ketamine, esketamine, has been increasingly used in clinical settings, which showed more potent effects than ketamine [60, 61] and has been shown to be effective in treatment-resistant patients with depression [62, 63]. Contrarily, increasing preclinical data suggest that arketamine, the (*R*)-enantiomer of ketamine, could produce greater potency and longer-lasting antidepressant-like actions in rodents than esketamine and that the side effects of arketamine are lower than those of esketamine and ketamine [55, 64, 65]. A single intravenous infusion of arketamine (0.5 mg/kg) is reported to produce rapid and sustained antidepressant actions in treatment-resistant patients with depression, and the side effects (i.e., psychotomimetic and dissociative effects) of arketamine (0.5 mg/kg) were much lower than those of esketamine (0.2 and 0.4 mg/kg) [63, 66]. Therefore, future research is needed to compare the efficacy of the perioperative application of esketamine and arketamine on postoperative depression.

The findings of the present meta-analysis should be interpreted with caution due to the following limitations, which are mostly related to the weaknesses of the original trials. First, the total sample size was relatively small, and heterogeneity in most analyses was relatively high, which might be due to the fact that most studies included had various types of anesthesia and different administration methods and dosages, although corresponding subgroup analyses were performed. Second, there was a lack of standardization for patient selection and follow-up periods. The assessed time point of postoperative depression varied among studies. Hence, the precise time course of the effect of ketamine on postoperative depression could not be evaluated. Studies used for subgroup analyses with regard to midazolam premedication were limited but it is consistent with previous study [67] that midazolam can reduce the mental adverse effects of ketamine. Finally, the scales used to assess postoperative depression were different, which also increased the sources of heterogeneity. Therefore, higher quality RCTs with a larger sample size are needed to confirm whether perioperative application of ketamine could improve the symptoms of postoperative depression and reduce postoperative pain intensity.

## Conclusion

The current meta-analysis indicated that perioperative application of ketamine is effective for reducing postoperative depression scores and pain intensity. However, ketamine increases the risk of nausea and vomiting, headache, hallucination, and dizziness compared with placebo, especially after a single-dose administration. In future clinical practice, the optimal approach for achieving the best antidepressant effect of ketamine with minimal adverse effects remains a major challenge.

### Supplementary information

Supplemental information
