# Supplementary material for: Efficacy and safety of perioperative application of ketamine on postoperative depression: A meta-analysis of randomized controlled studies
Source: Mol Psychiatry. 2023 Jan 20;28(6):2266–76. doi: 10.1038/s41380-023-01945-z (PMC10611576; doi:10.1038/s41380-023-01945-z)
Supplement: Supplementary file 1 — Supplemental information [file 41380_2023_1945_MOESM1_ESM.docx]

**Supplemental Information**

**Efficacy and safety of perioperative application of ketamine on postoperative depression: A meta-analysis of randomized controlled studies**

**Jie Guo^1#^, Di Qiu^2#^, Han-wen Gu^2^, Xing-ming Wang^2,3^, Kenji Hashimoto^3*^, Guang-fen Zhang^4*^, and Jian-jun Yang^2*^**

^1^Department of Anesthesiology, Shandong Cancer Hospital Affiliated to Shandong First Medical University, Jinan, China.

^2^Department of Anesthesiology, Pain and Perioperative Medicine, The first Affiliated Hospital of Zhengzhou University, Zhengzhou, Henan, China

^3^Division of Clinical Neuroscience, Chiba University Center for Forensic Mental Health, Chiba 260-8670, Japan

^4^Department of Anesthesiology, Shandong Provincial Hospital Affiliated to Shandong First Medical University, Jinan, China

^#^These authors contributed equally to this work.

*Corresponding Authors:

**
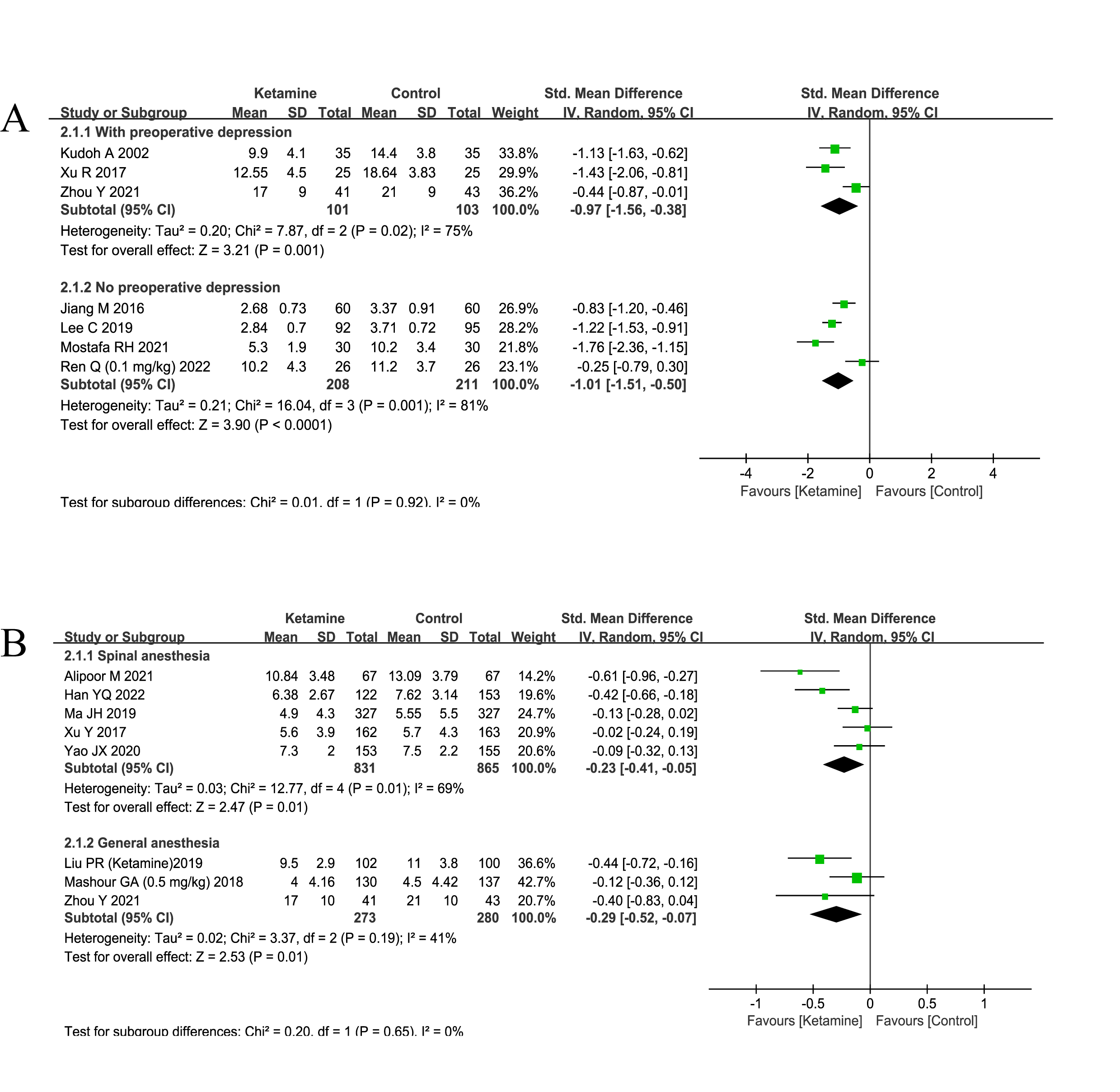
**

**Figure S1. Forest plots of subgroup analyses**

**A**. Subgroup analysis for postoperative depression rating scale according to preoperative depression (with or without). **B**. Subgroup analysis for postoperative depression rating scale according to anesthesia type (spinal anesthesia vs. general anesthesia) on postoperative day 1. CI, confidence interval. df, degrees of freedom.

**
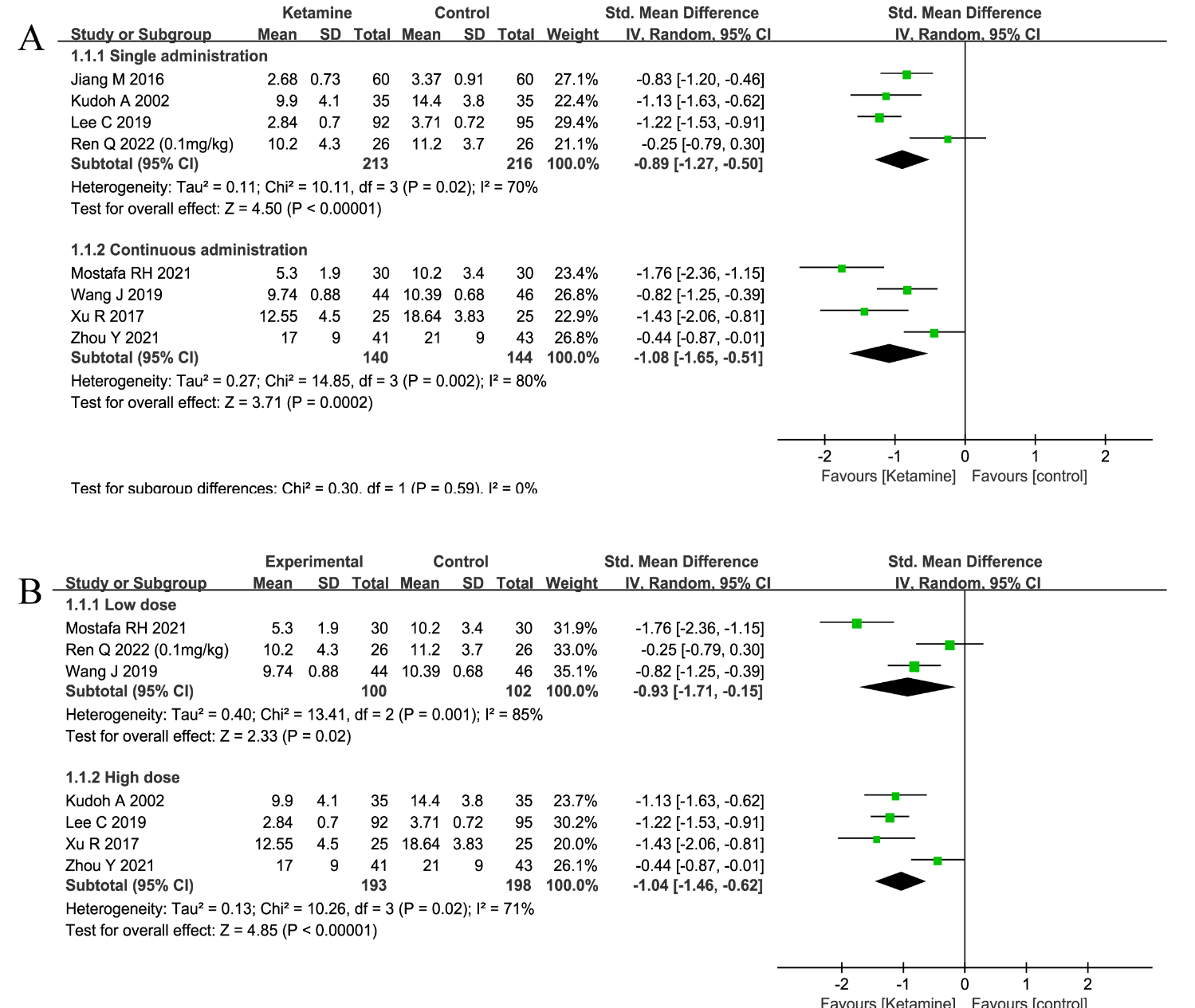
**

**Figure S2. Forest plots of subgroup analyses**

**A**. Subgroup analysis for the postoperative depression rating scale according to administration method of ketamine (single-dose administration vs. continued infusion administration). **B**. Subgroup analysis for postoperative depression rating scale according to dose of ketamine (low dose vs. high dose) on postoperative day 1. CI, confidence interval. df, degrees of freedom.


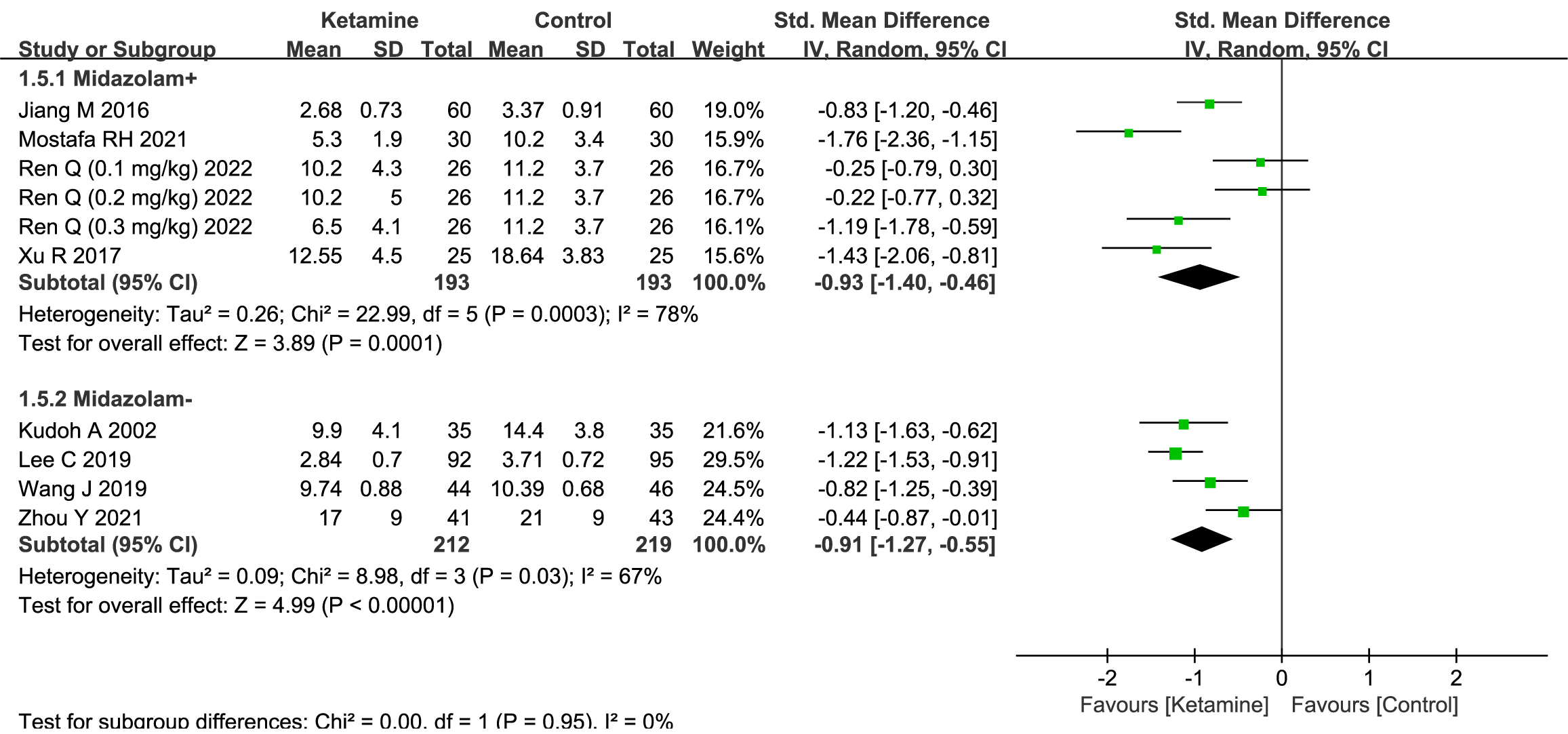


**Figure S3. Forest plots of subgroup analysis for postoperative depression rating scale according to with or without midazolam premedication**

CI, confidence interval. df, degrees of freedom.

**
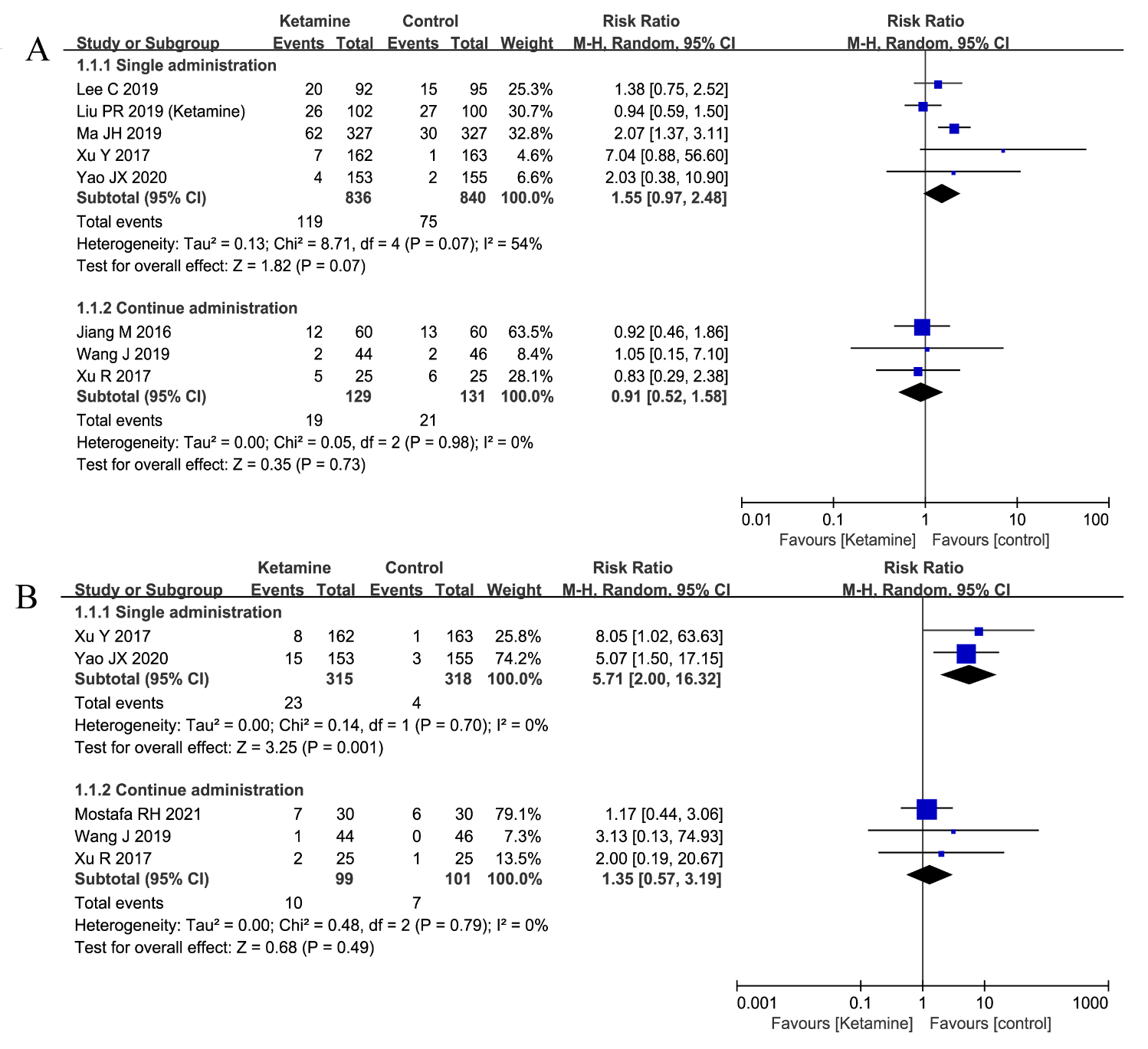
**

**Figure S4. Forest plots of subgroup analyses for adverse effects according to ketamine administration (single-dose vs. continued infusion)**

**A**. The risk of nausea and vomiting in the ketamine group and controls after single-dose administration and continued infusion of ketamine. **B**. The risk of headache in the ketamine group and controls after single-dose administration and continued infusion of ketamine. CI, confidence interval. df, degrees of freedom.

**
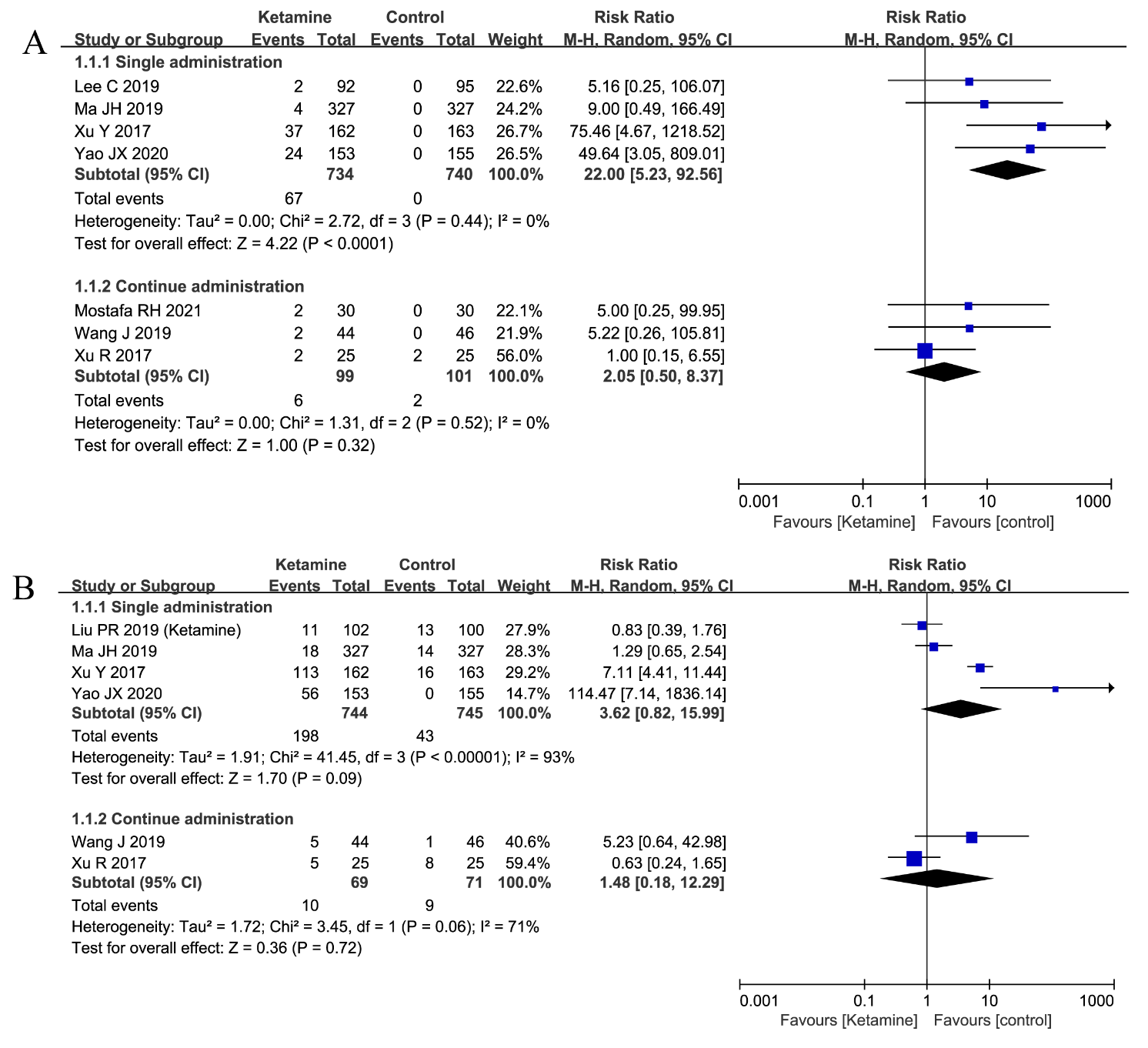
**

**Figure S5. Forest plots of subgroup analyses for adverse effects according to ketamine administration (single-dose administration vs. continued infusion administration)**

**A**. The risk of hallucinations in the ketamine group and controls after single-dose administration and continued infusion administration of ketamine. **B**. The risk of dizziness in the ketamine group and controls after single-dose administration and continued infusion administration of ketamine. CI, confidence interval. df, degrees of freedom.


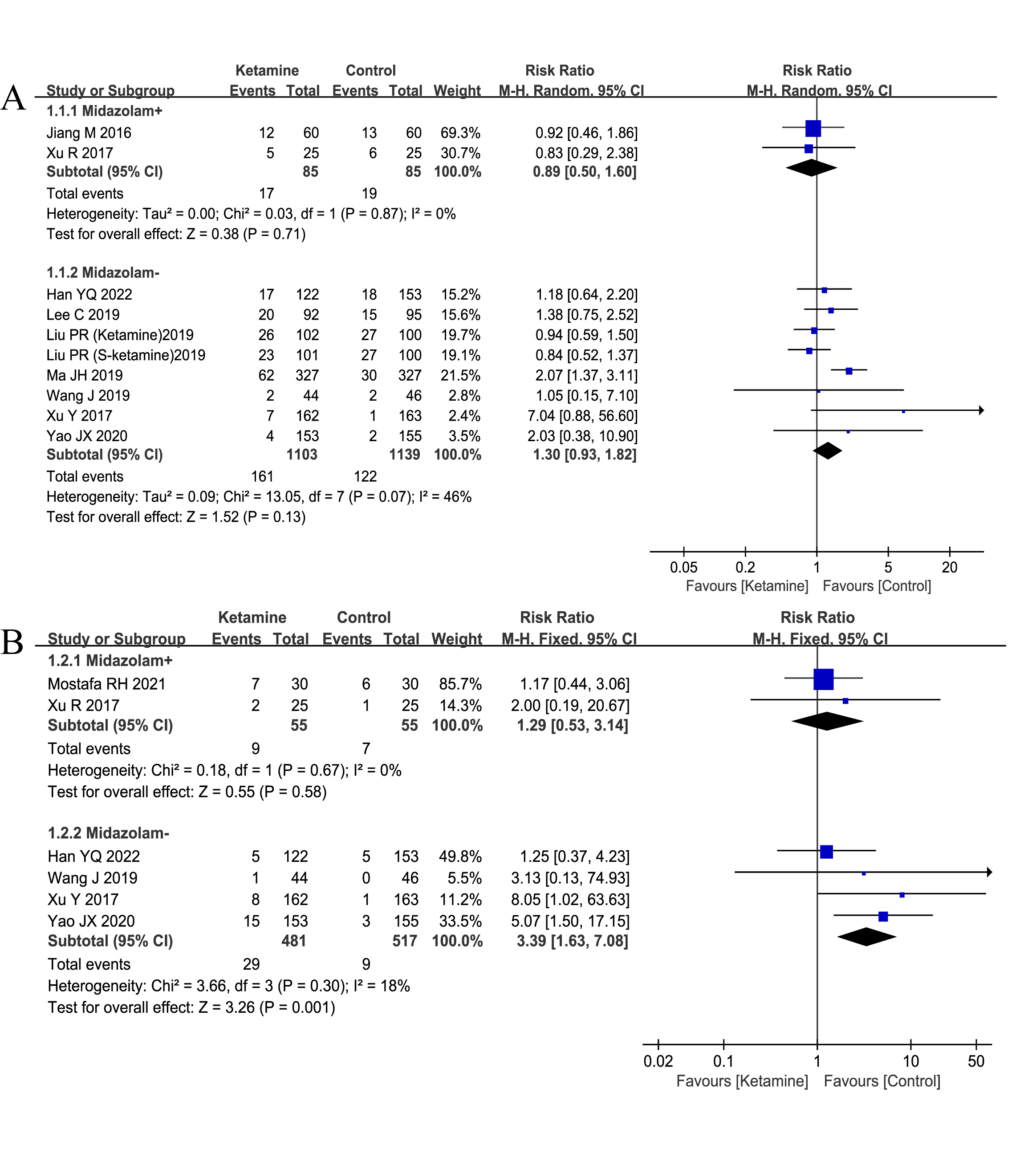


**Figure S6. Forest plots of subgroup analyses for adverse effects according to midazolam premedication (with vs. without)**

**A**. The risk of nausea and vomiting in the ketamine group and controls with or without midazolam premedication. **B**. The risk of headache in the ketamine group and controls with or without midazolam premedication. CI, confidence interval. df, degrees of freedom.


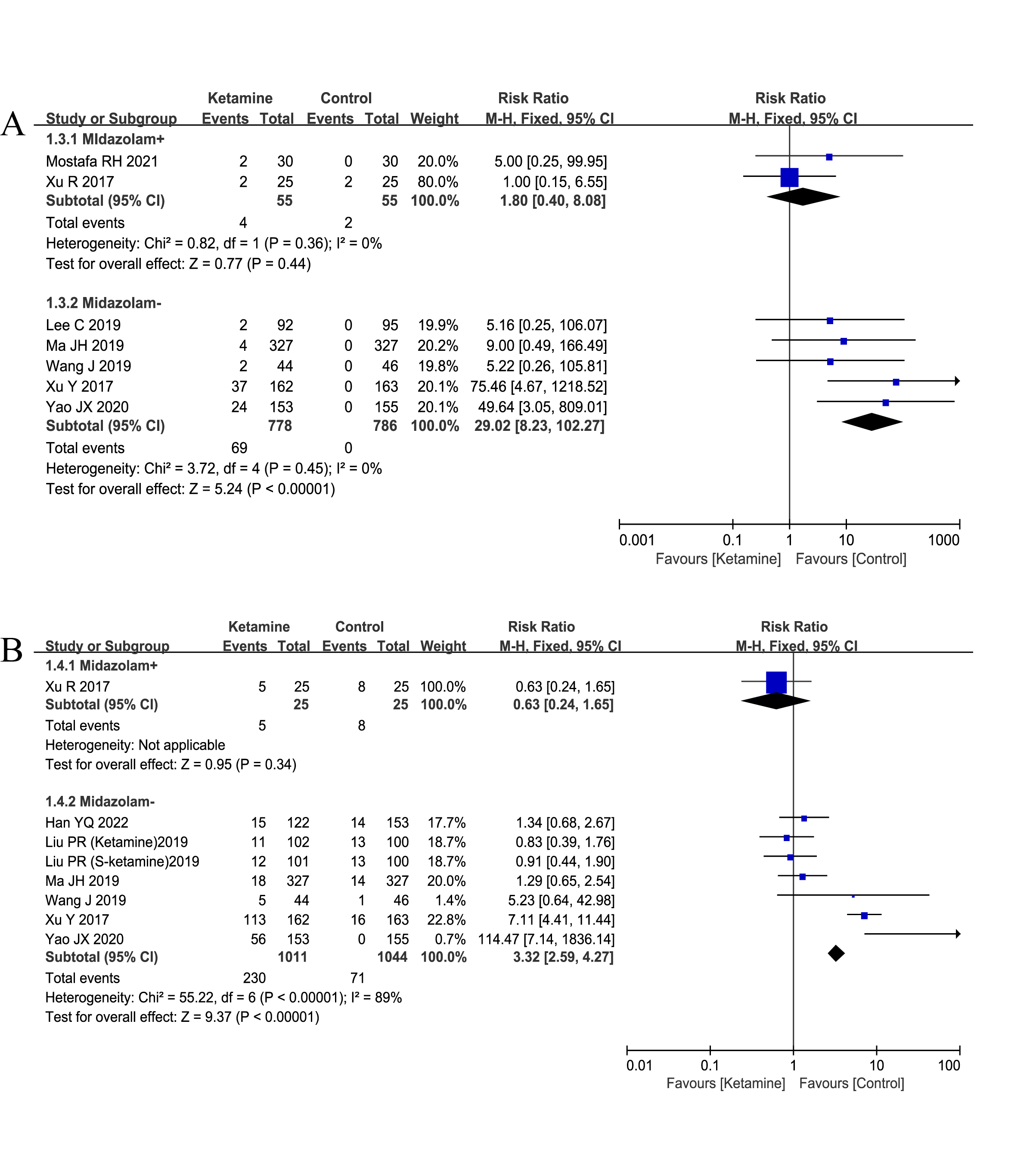


**Figure S7. Forest plots of subgroup analyses for adverse effects according to midazolam premedication (with vs. without)**

**A**. The risk of hallucinations in the ketamine group and controls with or without midazolam premedication. **B**. The risk of dizziness in the ketamine group and control group with or without midazolam premedication. CI, confidence interval. df, degrees of freedom.


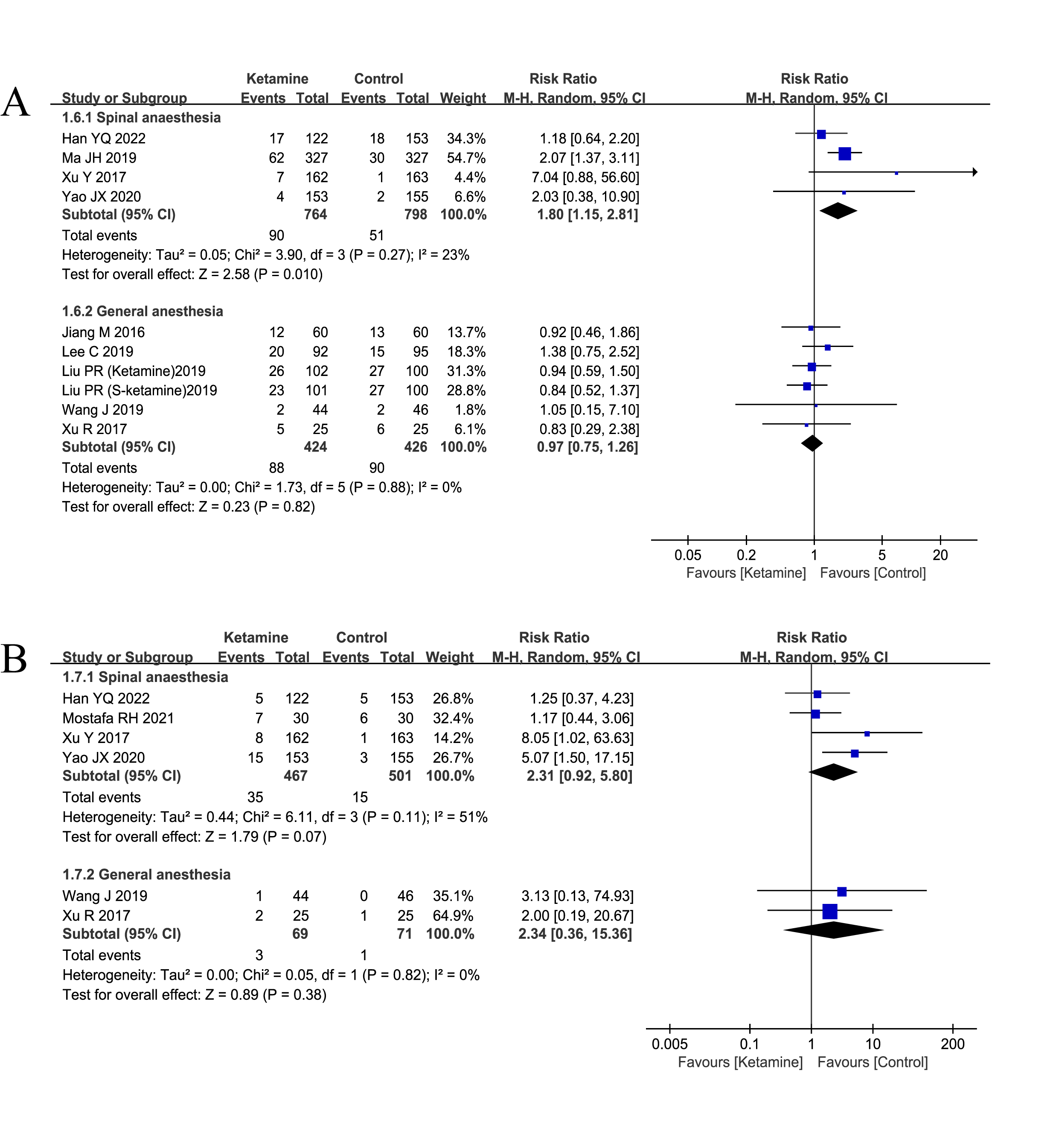


**Figure S8. Forest plots of subgroup analyses for adverse effects according to spinal anesthesia or general anesthesia**

**A**. The risk of nausea and vomiting in the ketamine group and controls with spinal anesthesia or general anesthesia. **B**. The risk of headache in the ketamine group and controls with spinal anesthesia or general anesthesia. CI, confidence interval. df, degrees of freedom.


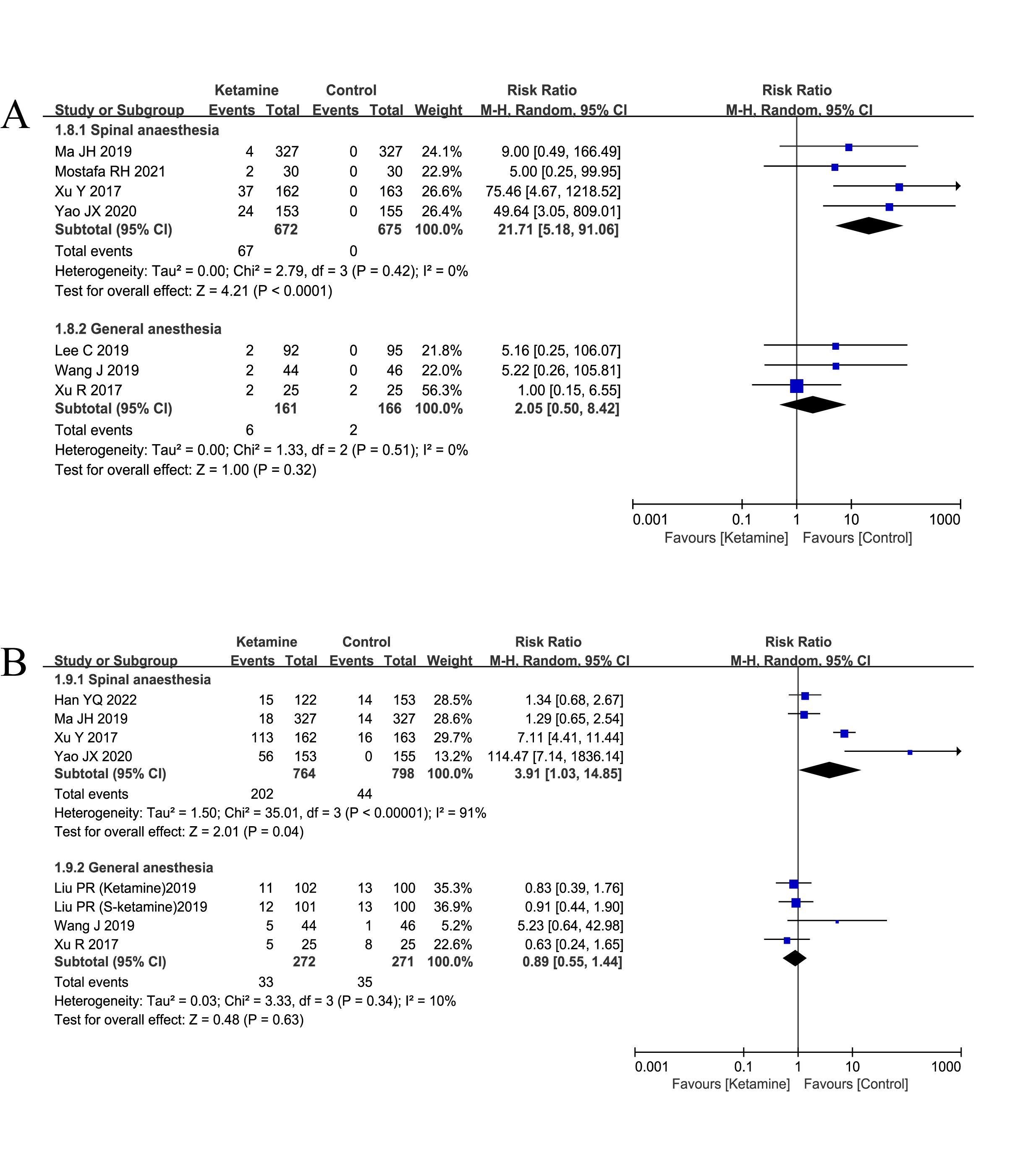


**Figure S9. Forest plots of subgroup analyses for adverse effects according to spinal anesthesia or general anesthesia**

**A**. The risk of hallucinations in the ketamine group and controls with spinal anesthesia or general anesthesia. **B**. The risk of dizziness in the ketamine group and controls with spinal anesthesia or general anesthesia. CI, confidence interval. df, degrees of freedom.
